# (1S*,2S*)-1,2-Di-*tert*-butyl­glycol

**DOI:** 10.1107/S1600536808043560

**Published:** 2008-12-24

**Authors:** Tobias Kerscher, Richard Betz, Peter Klüfers, Peter Mayer

**Affiliations:** a Ludwig-Maximilians Universität, Department Chemie und Biochemie, Butenandtstrasse 5–13 (Haus D), 81377 München, Germany

## Abstract

In the crystal structure of the title compound, C_10_H_22_O_2_, co-operative chains of O—H⋯O hydrogen bonds are established by intra- as well as inter­molecular inter­actions. These hydrogen bonds connect the mol­ecules into infinite strands along [100], with a binary level graph-set descriptor *C*_2_^2^(4). Excluding the H atoms on the hydr­oxy groups, the mol­ecule shows non-crystallographic *C*_2_ symmetry.

## Related literature

The compound was synthesized according to a published procedure (Boehrer *et al.*, 1997 ▶). For the crystal structures of other ethane-1,2-diol derivatives with bulky substituents, see: Betz & Klüfers (2007 ▶); Allscher *et al.* (2008 ▶). For graph-set descriptors, see: Etter *et al.* (1990 ▶); Bernstein *et al.* (1995 ▶). 
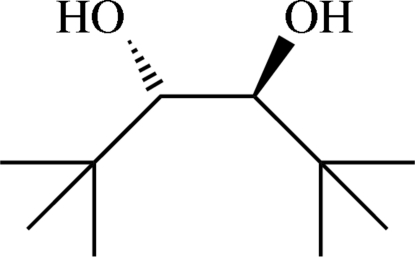


## Experimental

### 

#### Crystal data

C_10_H_22_O_2_
*M* *_r_* = 174.28
Orthorhombic, 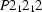

*a* = 9.7799 (3) Å
*b* = 16.3879 (7) Å
*c* = 6.9771 (3) Å
*V* = 1118.23 (8) Å^3^
*Z* = 4
Mo *K*α radiation
μ = 0.07 mm^−1^
*T* = 200 (2) K
0.30 × 0.09 × 0.02 mm

#### Data collection

Nonius KappaCCD diffractometer
Absorption correction: none
8640 measured reflections
1490 independent reflections
1253 reflections with *I* > 2σ(*I*)
*R*_int_ = 0.040

#### Refinement

*R*[*F* ^2^ > 2σ(*F* ^2^)] = 0.041
*wR*(*F* ^2^) = 0.109
*S* = 1.05
1490 reflections
117 parameters
H-atom parameters constrained
Δρ_max_ = 0.15 e Å^−3^
Δρ_min_ = −0.15 e Å^−3^

### 

Data collection: *COLLECT* (Nonius, 2004 ▶); cell refinement: *SCALEPACK* (Otwinowski & Minor, 1997 ▶); data reduction: *DENZO* (Otwinowski & Minor, 1997 ▶) and *SCALEPACK*; program(s) used to solve structure: *SIR97* (Altomare *et al.*, 1999 ▶); program(s) used to refine structure: *SHELXL97* (Sheldrick, 2008 ▶); molecular graphics: *ORTEP-3* (Farrugia, 1997 ▶); software used to prepare material for publication: *SHELXL97*.

## Supplementary Material

Crystal structure: contains datablocks global, I. DOI: 10.1107/S1600536808043560/bi2332sup1.cif

Structure factors: contains datablocks I. DOI: 10.1107/S1600536808043560/bi2332Isup2.hkl

Additional supplementary materials:  crystallographic information; 3D view; checkCIF report

## Figures and Tables

**Table 1:** Hydrogen-bond geometry (Å, °)

*D*—H⋯*A*	*D*—H	H⋯*A*	*D*⋯*A*	*D*—H⋯*A*
O1—H811⋯O2^i^	0.84	1.93	2.7721 (16)	176
O2—H821⋯O1	0.84	1.97	2.5129 (16)	121

Symmetry code: (i) 
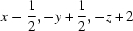
.

**Fig. 1.**
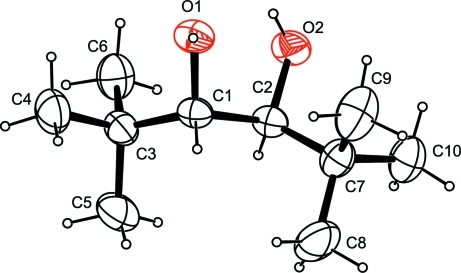
The molecular structure of the title compound, with atom labels and anisotropic displacement ellipsoids (drawn at 50% probability level) for non-H atoms.

**Fig. 2.**
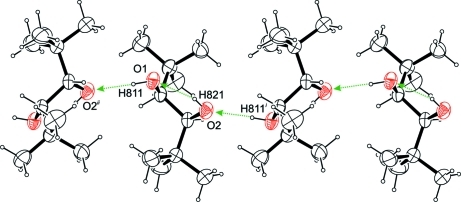
Hydrogen bonds in the crystal structure of the title compound, viewed along [0 0 1]. Underlying hydrogen bonds are not illustrated for clarity. Symmetry codes: ix + 1/2, -y + 1/2, -z + 2; iix - 1/2, -y + 1/2, -z + 2.

**Fig. 3.**
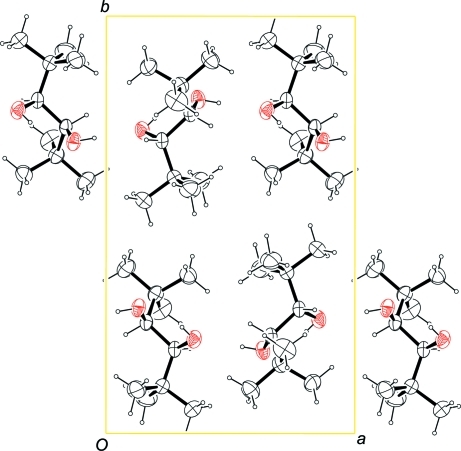
The packing of the title compound, viewed along [0 0 - 1].

## supplementary crystallographic information

### Comment

The title compound was synthesized as a potential chelating ligand with sterically demanding substituents on the carbon backbone to estimate the influence of steric pretense on coordination reactions with different central atoms.

In the molecule (Fig. 1) bond lengths and angles are found in the range apparent for other vicinal diols bearing sterically more demanding substituents (Betz & Klüfers, 2007, Allscher *et al.*, 2008). While the *tert*-butyl groups adopt a staggered conformation with respect to the hydroxy groups, the O atoms are present in a nearly eclipsed arrangement. The reason for this unfavourable conformation becomes evident when examining intermolecular contacts.

In the crystal structure, inter- and intramolecular hydrogen bonds are present which connect the molecules into strands along [1 0 0] (Fig. 2). The bulky hydrophobic *tert*-butyl groups encase this strand of hydroxyl groups. The hydrogen bonds form cooperative chains. In terms of graph-set analysis (Etter *et al.*, 1990; Bernstein *et al.*, 1995) the descriptor for these chains on the binary level is C^2^_2_(4).

Excluding the H atoms on the hydroxy groups, the molecule shows non-crystallographic *C*_2_ symmetry.

The molecular packing of the title compound is shown in Figure 3.

### Experimental

The title compound was prepared according to a published procedure (Boehrer *et al.*, 1997). Crystals suitable for X-ray studies were obtained upon recrystallization from boiling toluene.

### Refinement

Due to the absence of a strong anomalous scatterer, the absolute structure parameter, which is 0.982 with an estimated standard deviation of 1.241 for the unmerged data set, is meaningless. Thus 1056 Friedel opposites have been merged and the absolute configuration has been arbitrarily chosen.

Carbon-bound as well as oxygen-bound H atoms were placed in calculated positions (C—H 1.00 Å for CH-groups, C—H 0.98 Å for methyl groups and O—H 0.84 Å for hydroxy groups) and were included in the refinement in the riding model approximation, with *U*(H) set to 1.2*U*_eq_(C) for the CH-groups and 1.5*U*_eq_(C) for methyl groups and 1.5*U*_eq_(O) for the hydroxy groups.

### Crystal data

**Table:**

C_10_H_22_O_2_	*F*(000) = 392
*M**_r_* = 174.28	*D*_x_ = 1.035 Mg m^−^^3^
Orthorhombic, *P*2_1_2_1_2	Mo *K*α radiation, λ = 0.71073 Å
Hall symbol: P 2 2ab	Cell parameters from 13560 reflections
*a* = 9.7799 (3) Å	θ = 3.1–27.5°
*b* = 16.3879 (7) Å	µ = 0.07 mm^−^^1^
*c* = 6.9771 (3) Å	*T* = 200 K
*V* = 1118.23 (8) Å^3^	Rod, colourless
*Z* = 4	0.30 × 0.09 × 0.02 mm

### Data collection

**Table:**

Nonius KappaCCD diffractometer	1253 reflections with *I* > 2σ(*I*)
Radiation source: rotating anode	*R*_int_ = 0.040
MONTEL, graded multilayered X-ray optics	θ_max_ = 27.5°, θ_min_ = 3.2°
CCD; rotation images; thick slices scans	*h* = −12→11
8640 measured reflections	*k* = −19→21
1490 independent reflections	*l* = −9→8

### Refinement

**Table:**

Refinement on *F*^2^	Primary atom site location: structure-invariant direct methods
Least-squares matrix: full	Secondary atom site location: difference Fourier map
*R*[*F*^2^ > 2σ(*F*^2^)] = 0.041	Hydrogen site location: inferred from neighbouring sites
*wR*(*F*^2^) = 0.109	H-atom parameters constrained
*S* = 1.05	*w* = 1/[σ^2^(*F*_o_^2^) + (0.0621*P*)^2^ + 0.0637*P*] where *P* = (*F*_o_^2^ + 2*F*_c_^2^)/3
1490 reflections	(Δ/σ)_max_ < 0.001
117 parameters	Δρ_max_ = 0.15 e Å^−^^3^
0 restraints	Δρ_min_ = −0.15 e Å^−^^3^

### Fractional atomic coordinates and isotropic or equivalent isotropic displacement parameters (Å^2^)

**Table:**

	*x*	*y*	*z*	*U*_iso_*/*U*_eq_	
O1	0.13030 (11)	0.30082 (8)	0.96003 (18)	0.0402 (3)	
H811	0.0476	0.2922	0.9857	0.060*	
O2	0.35411 (12)	0.22582 (8)	0.9762 (2)	0.0437 (4)	
H821	0.3054	0.2548	1.0484	0.066*	
C1	0.16231 (16)	0.26595 (10)	0.7782 (2)	0.0330 (4)	
H1	0.0792	0.2371	0.7292	0.040*	
C2	0.27611 (16)	0.20174 (10)	0.8121 (2)	0.0332 (4)	
H2	0.3386	0.2035	0.6988	0.040*	
C3	0.19990 (19)	0.33399 (11)	0.6353 (3)	0.0393 (4)	
C4	0.0811 (2)	0.39538 (13)	0.6286 (3)	0.0551 (6)	
H41	0.0731	0.4228	0.7530	0.083*	
H42	−0.0042	0.3665	0.6001	0.083*	
H43	0.0987	0.4360	0.5285	0.083*	
C5	0.2176 (3)	0.29796 (15)	0.4358 (3)	0.0628 (6)	
H51	0.2281	0.3422	0.3424	0.094*	
H52	0.1370	0.2653	0.4031	0.094*	
H53	0.2992	0.2632	0.4335	0.094*	
C6	0.3298 (2)	0.37869 (13)	0.6961 (3)	0.0516 (5)	
H61	0.4075	0.3409	0.6917	0.077*	
H62	0.3188	0.3994	0.8270	0.077*	
H63	0.3466	0.4244	0.6087	0.077*	
C7	0.22584 (18)	0.11294 (12)	0.8338 (3)	0.0409 (5)	
C8	0.1634 (3)	0.08512 (14)	0.6441 (4)	0.0687 (7)	
H81	0.2274	0.0967	0.5394	0.103*	
H82	0.0776	0.1145	0.6218	0.103*	
H83	0.1453	0.0264	0.6494	0.103*	
C9	0.1230 (2)	0.10514 (15)	0.9965 (4)	0.0656 (7)	
H91	0.0986	0.0476	1.0138	0.098*	
H92	0.0407	0.1366	0.9653	0.098*	
H93	0.1634	0.1262	1.1150	0.098*	
C10	0.3495 (2)	0.05872 (12)	0.8749 (4)	0.0542 (5)	
H101	0.3896	0.0740	0.9985	0.081*	
H102	0.4176	0.0658	0.7733	0.081*	
H103	0.3204	0.0015	0.8793	0.081*	

### Atomic displacement parameters (Å^2^)

**Table:**

	*U*^11^	*U*^22^	*U*^33^	*U*^12^	*U*^13^	*U*^23^
O1	0.0321 (6)	0.0536 (8)	0.0349 (6)	0.0056 (6)	0.0041 (5)	−0.0067 (6)
O2	0.0344 (6)	0.0518 (8)	0.0448 (7)	0.0046 (5)	−0.0136 (6)	−0.0073 (6)
C1	0.0260 (7)	0.0416 (9)	0.0314 (8)	0.0013 (7)	−0.0020 (7)	−0.0047 (7)
C2	0.0251 (7)	0.0428 (9)	0.0317 (8)	0.0007 (7)	−0.0004 (6)	−0.0032 (7)
C3	0.0397 (9)	0.0446 (10)	0.0334 (9)	0.0051 (8)	−0.0020 (8)	0.0026 (8)
C4	0.0548 (11)	0.0537 (12)	0.0568 (12)	0.0140 (10)	−0.0059 (11)	0.0094 (11)
C5	0.0841 (15)	0.0716 (15)	0.0327 (10)	0.0129 (13)	0.0028 (10)	0.0025 (10)
C6	0.0452 (11)	0.0493 (11)	0.0602 (13)	−0.0051 (9)	0.0029 (10)	0.0121 (10)
C7	0.0341 (9)	0.0374 (9)	0.0513 (11)	0.0025 (7)	0.0012 (8)	−0.0015 (8)
C8	0.0733 (15)	0.0485 (12)	0.0843 (18)	−0.0004 (12)	−0.0263 (14)	−0.0208 (12)
C9	0.0580 (13)	0.0499 (12)	0.0889 (18)	−0.0016 (10)	0.0295 (12)	0.0095 (12)
C10	0.0463 (10)	0.0435 (11)	0.0729 (14)	0.0087 (9)	−0.0010 (11)	0.0023 (11)

### Geometric parameters (Å, °)

**Table:**

O1—C1	1.426 (2)	C5—H53	0.980
O1—H811	0.840	C6—H61	0.980
O2—C2	1.431 (2)	C6—H62	0.980
O2—H821	0.840	C6—H63	0.980
C1—C3	1.540 (3)	C7—C9	1.522 (3)
C1—C2	1.550 (2)	C7—C8	1.527 (3)
C1—H1	1.000	C7—C10	1.528 (3)
C2—C7	1.543 (3)	C8—H81	0.980
C2—H2	1.000	C8—H82	0.980
C3—C5	1.522 (3)	C8—H83	0.980
C3—C6	1.527 (3)	C9—H91	0.980
C3—C4	1.538 (2)	C9—H92	0.980
C4—H41	0.980	C9—H93	0.980
C4—H42	0.980	C10—H101	0.980
C4—H43	0.980	C10—H102	0.980
C5—H51	0.980	C10—H103	0.980
C5—H52	0.980		
			
C1—O1—H811	109.5	H52—C5—H53	109.5
C2—O2—H821	109.5	C3—C6—H61	109.5
O1—C1—C3	109.77 (14)	C3—C6—H62	109.5
O1—C1—C2	107.08 (13)	H61—C6—H62	109.5
C3—C1—C2	114.77 (13)	C3—C6—H63	109.5
O1—C1—H1	108.3	H61—C6—H63	109.5
C3—C1—H1	108.3	H62—C6—H63	109.5
C2—C1—H1	108.3	C9—C7—C8	110.91 (18)
O2—C2—C7	110.58 (14)	C9—C7—C10	109.53 (17)
O2—C2—C1	108.52 (13)	C8—C7—C10	107.79 (17)
C7—C2—C1	115.24 (14)	C9—C7—C2	111.26 (16)
O2—C2—H2	107.4	C8—C7—C2	108.91 (16)
C7—C2—H2	107.4	C10—C7—C2	108.35 (14)
C1—C2—H2	107.4	C7—C8—H81	109.5
C5—C3—C6	110.22 (19)	C7—C8—H82	109.5
C5—C3—C4	108.20 (17)	H81—C8—H82	109.5
C6—C3—C4	108.86 (16)	C7—C8—H83	109.5
C5—C3—C1	109.78 (16)	H81—C8—H83	109.5
C6—C3—C1	111.47 (15)	H82—C8—H83	109.5
C4—C3—C1	108.22 (14)	C7—C9—H91	109.5
C3—C4—H41	109.5	C7—C9—H92	109.5
C3—C4—H42	109.5	H91—C9—H92	109.5
H41—C4—H42	109.5	C7—C9—H93	109.5
C3—C4—H43	109.5	H91—C9—H93	109.5
H41—C4—H43	109.5	H92—C9—H93	109.5
H42—C4—H43	109.5	C7—C10—H101	109.5
C3—C5—H51	109.5	C7—C10—H102	109.5
C3—C5—H52	109.5	H101—C10—H102	109.5
H51—C5—H52	109.5	C7—C10—H103	109.5
C3—C5—H53	109.5	H101—C10—H103	109.5
H51—C5—H53	109.5	H102—C10—H103	109.5
			
O1—C1—C2—O2	−28.26 (17)	O1—C1—C3—C4	−55.45 (18)
C3—C1—C2—O2	93.86 (17)	C2—C1—C3—C4	−176.09 (15)
O1—C1—C2—C7	96.34 (17)	O2—C2—C7—C9	66.46 (19)
C3—C1—C2—C7	−141.55 (15)	C1—C2—C7—C9	−57.1 (2)
O1—C1—C3—C5	−173.34 (16)	O2—C2—C7—C8	−170.99 (15)
C2—C1—C3—C5	66.0 (2)	C1—C2—C7—C8	65.5 (2)
O1—C1—C3—C6	64.23 (18)	O2—C2—C7—C10	−54.00 (19)
C2—C1—C3—C6	−56.4 (2)	C1—C2—C7—C10	−177.51 (16)

### Hydrogen-bond geometry (Å, °)

**Table:**

*D*—H···*A*	*D*—H	H···*A*	*D*···*A*	*D*—H···*A*
O1—H811···O2^i^	0.84	1.93	2.7721 (16)	176
O2—H821···O1	0.84	1.97	2.5129 (16)	121

Symmetry codes: (i) *x*−1/2, −*y*+1/2, −*z*+2.
